# 5-fluorouracil metabolism monitored in vivo by 19F NMR.

**DOI:** 10.1038/bjc.1984.146

**Published:** 1984-07

**Authors:** A. N. Stevens, P. G. Morris, R. A. Iles, P. W. Sheldon, J. R. Griffiths


*Br. J. Cancer* (1984), **50**, 113–117

### Short Communication

### 5-Flourouracil metabolism monitored *in vivo* by $^{19}$F NMR

A.N. Stevens[1], P.G. Morris[2], R.A. Iles[3], P.W. Sheldon[4] & J.R. Griffiths[1]

[1]*Department of Biochemistry, St George's Hospital Medical School, Cranmer Terrace, London SW17 0RE;* [2]*MRC Biomedical NMR Centre, National Institute for Medical Research, London NW7 1AA;* [3]*Academic Unit of Metabolism and Endocrinology, Alexandra Wing, London Hospital Medical College, London E1 1BB; and* [4]*Department of Physics, Institute of Cancer Research, Clifton Avenue, Sutton, Surrey SM2 5PX, UK.*

The nuclear magnetic resonance (NMR) method makes it possible to analyse the chemical constituents of living animals or humans repeatedly and non-invasively. It has mainly been applied to the study of endogenous phosphorylated or carbon containing compounds (Gadian, 1982; Iles *et al.*, 1982) but could equally be used to trace the metabolic fate of exogenously administered substances such as drugs. At present it is necessary to kill many animals in order to determine the fate of a drug at its target site (or at the sites where it is detoxified) and it is almost impossible to obtain such information in humans. By using new *in vivo* NMR techniques it should be possible to follow the metabolic fate of a drug either in an animal or patient. The most favourable atomic nucleus for studies of this kind is $^{19}$F: there is no background from endogenous compounds, $^{19}$F gives intense NMR signals with a wide chemical shift range, and many fluorinated drugs are in clinical use. In this paper we describe the use of $^{19}$F NMR to monitor the metabolism of 5-fluorouracil in tumours and in the liver.

The fluorinated pyrimidines, 5-fluorouracil (5FU), 5-fluorouridine (FUrd) and 5-fluoro-2-deoxyuridine (FdU) are widely used in the treatment of disseminated human cancers, especially of the gastrointestinal tract, breast and ovary (Martindale, 1982). The metabolism of these drugs has been extensively studied and it is clear that with a few important exceptions they participate in the same pathways as uracil and its metabolites (Figure 1). Treatment with fluorinated pyrimidines produces two major effects in cells: (i) inhibition of DNA synthesis by inhibition of dTMP synthetase (EC 2.1.1.41) by fluorodeoxyuridine monophosphate (FdUMP) (Heidelberger, 1974); and (ii) alteration in the processing and function of some types of RNA because of extensive incorporation of 5FU in place of uracil (Heidelberger, 1974; Carrico & Glazer, 1979). Which of these effects accounts for the major antitumour property of these drugs is a matter of contention.

Recent studies have been directed at developing combined chemotherapeutic regimens which incorporate the fluorinated pyrimidines with drugs such as methotrexate, thymidine or PALA (Goulian *et al.*, 1980; Bedikian *et al.*, 1981; Au *et al.*, 1982) or developing new analogues (Sakurai, 1981). In both cases the aim is to alter the rate and fate of metabolism of the drug and hence increase its therapeutic efficacy.

Major problems in this area are the provision of precise methods with the ability to quantify all aspects of metabolism of the drug: its metabolites are unstable and difficult to detect. Pogolotti *et al.* (1981) and Sommadossi *et al.* (1982) have overcome many of these problems, but conventional analysis of uptake into solid tumour models has a number of difficulties, not least in satisfactory removal of the tissue. Studies on 5FU metabolism in cell culture cannot give information about the uptake or fate of the drug in an intact tumour (Pogolotti *et al.*, 1981), while plasma pharmacodynamics give at best only a very indirect assessment of a drug's efficacy (Cano *et al.*, 1981).

We were able to follow the metabolism of i.v. injected 5FU *in situ* both in implanted tumours and livers of C57 mice by $^{19}$F-NMR using surface coils.

Livers were examined in either female C57 or C57Bl/Cbi mice. Lewis lung carcinomas were implanted in female C57Bl/Cbi mice by s.c. injection of $2.5 \times 10^4$ viable tumour cells (obtained by trypsin/DNase digestion) over the sacral region and studied at a mean diameter of 6–7 mm. Animals were anaesthetised using sodium pentobarbitone $60\,mg\,kg^{-1}$ by i.p. injection. A $25\,mg\,ml^{-1}$ solution of 5FU (Roche Products) was then injected into the jugular vein. For NMR spectroscopy mice were secured vertically within a purpose built probe. By means of an abdominal incision a flat one turn 7 mm diameter radiofrequency (R.f.) coil was placed on the surface of the liver. To prevent evaporation and to electrically insulate the coil, the liver surface was covered with a thin plastic film. In the case of tumour-bearing mice the coil was laid over the

Correspondence: J.R. Griffiths
Received 23 January 1984; accepted 14 April 1984.

© The Macmillan Press Ltd., 1984

**Figure 1** The pathway of 5FU metabolism. 5FU (5-fluorouracil), FUR (fluorouracil ribose) FUMP, FUDP, FUTP (fluorouracil mono, di and triphosphate); FUdR (fluorouracil deoxyribose); FdUMP, FdUDP, FdUTP (fluorodeoxyuracil mono, di, triphosphate); $FUH_2$ (dihydrofluorouracil); FUPA (fluoro-β-ureidopropionic acid); Fβala (fluoro-β-alanine).

tumour. The probe was inserted into the magnet of a wide bore Bruker WM200 spectrometer. $^{19}F$ spectra were recorded at an irradiation frequency of 188MHz and a sweep width of $\pm 6000$ Hz with 4 K data points. Radiofrequency pulses of 5 μs pulse width with a recycle time of 0.5 s were used. For all spectra the line broadening was 12 Hz. Integration of NMR peaks was by the method of cut and weigh (Iles *et al.*, 1982). Each experiment was performed on three occasions with essentially identical results. The figures show the result of a typical experiment in each case.

The results (Figure 2) clarify a number of steps in the pathway of 5FU metabolism (Figure 1). Degradation of 5FU proceeds by reduction of the pyrimidine ring and then hydrolysis to fluoro-β-ureidopropionic acid (FUPA) which is subsequently hydrolysed to fluoro-β-alanine (Fβala), $NH_4^+$ and $CO_2$. From the spectra in Figure 2a it is clear that a small dose of 5FU is fully converted to dihydrofluorouracil ($FUH_2$) and Fβala within 30 min. Most of the $FUH_2$ is then converted to Fβala within a further 3 h. After higher doses of 5FU (180 mg $kg^{-1}$) a constant $FUH_2$ peak was seen throughout the experiment (Figure 2b), although a fall in 5FU and a rise in the Fβala peak was taking place. No conversion of 5FU to toxic nucleosides or nucleotides was observed in the liver, even at high dosages.

By contrast, tumour tissue degrades 5FU at a much slower rate and the catabolic products found in the liver were not detected. Low or high doses of 5FU were converted to deoxynucleotide and deoxynucleoside derivatives (Figure 2c and d). For reasons discussed in the legend to Figure 2 we were unable to resolve the mono-, di- and tri-phosphate derivatives so we have assigned this peak as FdUMP. In a control experiment a $^{19}F$ signal was not obtained from muscle in the sacral region.

Pogolotti *et al.* (1981) found that all the $^3H$-5FU taken up by cultured cells was tightly bound to proteins; 5FdUMP complexed in this manner would probably be invisible by NMR. In the high dose experiments (180 mg $kg^{-1}$) there was considerable conversion of 5FU to 5FdUMP (Figure 2d). This peak had a narrow linewidth (126 Hz) suggesting that the 5FdUMP was free in solution. In contrast, at the low dose (30 mg $kg^{-1}$), the formation of 5FdUMP was barely detectable, although the linewidth of its peak was consistent with a compound in a mobile environment. The weak signal from 5FdUMP suggests that most of the metabolite was in an environment invisible to NMR, perhaps bound to a protein.

It has been suggested that thymidine could be used to protect normal cells against 5FU toxicity while potentiating its antitumour effect. 5FU is thought to act by conversion to 5dUMP which inhibits thymidylate synthetase or by conversion to 5-FUTP which causes incorporation of 5FU into RNA. Thymidine has been found to enhance 5FU incorporation into RNA of some tumour cell lines but not into bone marrow or gut cells (Speigelman *et al.*, 1980).

The experiments we have carried out in which thymidine (180 mg $kg^{-1}$) has been administered

**Figure 2** Uptake of 5-fluorouracil (5FU) into liver and implants of Lewis lung tumour in C57 mice monitored by $^{19}$F NMR spectroscopy after the i.v. injection of 30 mg kg$^{-1}$ 5FU (a,c) and 180 mg kg$^{-1}$ 5FU (b,d) into the jugular vein. Peak assignments have been obtained from aqueous tissue extracts doped with tracer amounts of the suspected compounds. Times refer to time after injection of 5FU. Peak A (△) arises primarily from the conversion of 5FU to the major anabolic product FdUMP and probably contains contributions from the corresponding di- and tri-phosphates. When prepared from FdUMP by the action of nucleoside monophosphate kinase (13) both gave resonances shifted only 1 ppm upfield from FdUMP when dissolved in 100 μM TEA buffer pH 7.6.

Peak B (▲) corresponds to the peak of 5FdU and is only visible after concurrent thymidine therapy (Figure 3). Peak C (●, ○) is 5FU. Peak D (■, □) probably represents the first ring cleavage product of the 5FU catabolic pathway ($FUH_2$). Peak E (◆, ◇) corresponds to fluor-β-alanine (Fβala) the end product of 5FU metabolism. Chemical shift values have been derived from the assignment of 5FU and Fβala which provide non-titrable internal references. All shift values are given relative to a pure solution of fluorotryptophan.

simultaneously with 5FU (30 mg kg$^{-1}$) are consistent with the suggestion that thymidine enhances 5FU toxicity by competing with 5FU for catabolic pathway enzymes in the liver and so prolongs 5FU clearance (Kirkwood *et al.*, 1980). A second effect is the appearance of deoxyuracil derivatives; this is suprising as they are not seen after administration of high doses of 5FU alone. Thymidine is known to induce the synthesis of thymidine phosphorylase which catalyses the conversion of 5FU to the toxic 5FUdR (Ardalan & Glazer, 1981). The most probable assignment of peak B in Figure 3a is 5FUdR.

The titration curve of 5FuDR showed a pK of 7.5; thus FUdR could clearly serve as a probe for intracellular pH. When injected into control mice at a dose of 180 mg kg$^{-1}$ the 5FUdR gave a peak in the spectrum from liver with a chemical shift corresponding to an intracellular pH of 7.2 ± 0.05 ($n$ = 5). This compares with measurements of intracellular pH in a similar group of mice using the $^{31}$P-NMR signal of Pi of 7.4 ± 0.6, $P$ = 0.05 ($n$ = 5).

In order to estimate the sensitivity of the technique (and to determine the 5FU signal likely to arise from the extra-cellular space) we repeated the experiments using 5-fluoro[6-$^{14}$C]uracil at 30 mg kg$^{-1}$. There was a rapid elimination from the blood in the first 30 min followed by a slower phase (Figure 4a) whereas the accumulation of radioactivity in the liver reached a plateau at 60 min. A similar curve was observed when the total fluorine integrals of the liver spectra were measured. From these data we calculate the the minimum

**Figure 3** The uptake of 5FU into the liver of C57 mice monitored by $^{19}F$ NMR spectroscopy after injection of 5FU 30 mg kg$^{-1}$ + thymidine 180 mg kg$^{-1}$. Peak assignments are given in the legend for **Figure 2**.

concentration of 5FU observable in the current series of experiments by $^{19}F$ NMR was 0.5 $\mu$mol g$^{-1}$ wet wt. The NMR signal from 5FU in the blood and extracellular space of the liver (and also of the tumour) will clearly be negligible at this dose (Figure 4b).

$^{19}F$ NMR can provide a direct, non-invasive measurement of the metabolism of a fluorinated drug both at its site of action and at its site(s) of detoxification. The action of adjuvant drugs on its metabolism can readily be demonstrated.

NMR observations of tumours in patients are now possible (Griffiths *et al.*, 1983) so the present method could be extended to clinical medicine. In addition to basic studies on drug metabolism of the kind described above it should prove possible to obtain a non-invasive assay of a drug and its metabolites at its sites of action in each patient and this would clearly assist patient management. Although $^{19}F$ is especially favourable this method need not be restricted to this nucleus. $^{13}C$ labelling for example (Alger *et al.*, 1981) could be used in almost any drug.

This work was funded by the Cancer Research Campaign. We also thank the MRC Biomedical NMR Centre for the use of their facilities and Mrs V. Marvell for typing the manuscript.

**Figure 4** (a) The uptake of $^{14}C$ label into mouse liver after the injection of 30 mg kg$^{-1}$ 5FU containing 2.2 $\mu$Ci 5-fluoro-[6-$^{14}C$]uracil (Amersham International): (b) the elimination of $^{14}C$ label from the blood stream. Results are the means of 5 experiments in each case; bars indicate s.e. Livers were removed by freeze clamping, extracted in 10% TCA and neutralised with Tris base. Blood was taken from the vena cava and quenched in an equal volume of TCA. Aliquots were counted by liquid scintillation.

**References**

ALGER, J.R., SILLERUD, L.O., BEHAR, K.L. & 5 others. (1981). In vivo carbon-13 nuclear magnetic resonance studies of mammals. *Science* **214,** 660.

ARDALAN, B. & GLAZER, R. (1981). An update on the biochemistry of 5FU. *Cancer Treat. Rev.,* **8,** 157.

AU, J.L., YOUCEF, M.R., LEDESMA, E.J., MITTELMAN, A. & CREAVAN, P.J. (1982). Clinical pharmacological studies of concurrent infusion of 5-flourouracil and thymidine in treatment of colorectal carcinomas. *Cancer Res.,* **42,** 2930.

BEDIKIAN, A.Y., STROEHLEIN, J.R., KARLIN, D.A., BENNETTS, R.W., BODEY, G.P. & VALDIVIESO, M. (1981). Chemotherapy for colorectal cancer with a combination of PALA and 5FU. *Cancer Treat. Rep.,* **65,** 747.

CANO, J.P., AUBERT, J.P., RIGAULT, R. & 5 others. (1981). Advantages and limitations of pharmacokinetic studies in the rationalization of anticancer therapy: methotrexate and 5FU. *Cancer Treatment Reps.,* **65,** 33.

CARRICO, C.K. & GLAZER, R.I. (1979). Effect of 5FU on the synthesis and translation of polyadenylic acid-containing RNA from regenerating rat liver. *Cancer Res.,* **39,** 3694.

GADIAN, D.G. (1982). *NMR and Its Application to Living Systems,* Oxford University Press, Oxford.

GOULIAN, M., BLEILE, B. & TSENG, B.Y. (1980). Methotrexate-induced misincorporation of uracil into DNA. *Proc. Nat. Acad. Sci.,* **77,** 1956.

GRIFFITHS, J.R., CADY, E., EDWARDS, R.H.T., MCCREADY, V.R., WILKIE, D.R. & WILTSHAW, E. (1983). $^{31}$P NMR studies of a human tumour in situ. *Lancet,* **i,** 1435.

HEIDELBERGER, C. (1974). Fluorinated pyrimidines and their nucleosides. In: *Antineoplastic and Immunosuppressive Agents.* Vol. 38, p. 193. Eds. Sartorelli, A.C. & Johns, D.G. Springer Verlag, NY.

ILES, R.A., STEVENS, A.N. & GRIFFITHS, J.R. (1982). NMR studies of metabolites in living tissues. *Prog. NMR Spect.,* **15,** 49.

KIRKWOOD, J.M., ENSMINGER, W., ROSOWSKY, A., PAPATHANASOPOULOS, N. & FREI, E. (1980). Comparison of pharmacokinetics of 5FU and 5FU in concurrent thymidine infusions including a phase 1 trial. *Cancer Res.,* **40,** 107.

MARTINDALE (1982). *The Extra Phamacopoeia,* 28th Edn. Ed. J.E.F. Reynolds, Pharmaceutical Press, London.

POGOLOTTI, A.L., NOLAN, P.A. & SANTI, D.V. (1981). Methods for the complete analysis of 5-fluorouracil metabolites in cell extracts. *Anal. Biochem.,* **117,** 178.

SAKURAI, Y. (1981). Studies of analogs of fluorinated pyrimidine in Japan. *Recent Results Cancer Res.,* **76,** 91.

SOMMADOSSI, J.P., GEWIRTZ, D.A., DIASIO, R.B., AUBERT, C., CANO, J.P. & GOLDMAN, I.D. (1982). Rapid catabolism of 5-FU in freshly isolated rat hepatocytes as analysed by HPLC. *J. Biol. Chem.,* **257,** 8171.

SPEIGELMAN, S., NAYAK, R., SAWYER, R., STOLFI, R. & MARTIN, D. (1980). Potentiation of the antitumour activity of 5FU by thymidine and its correlation with the formation of (5FU) RNA. *Cancer,* **45,** 1129.


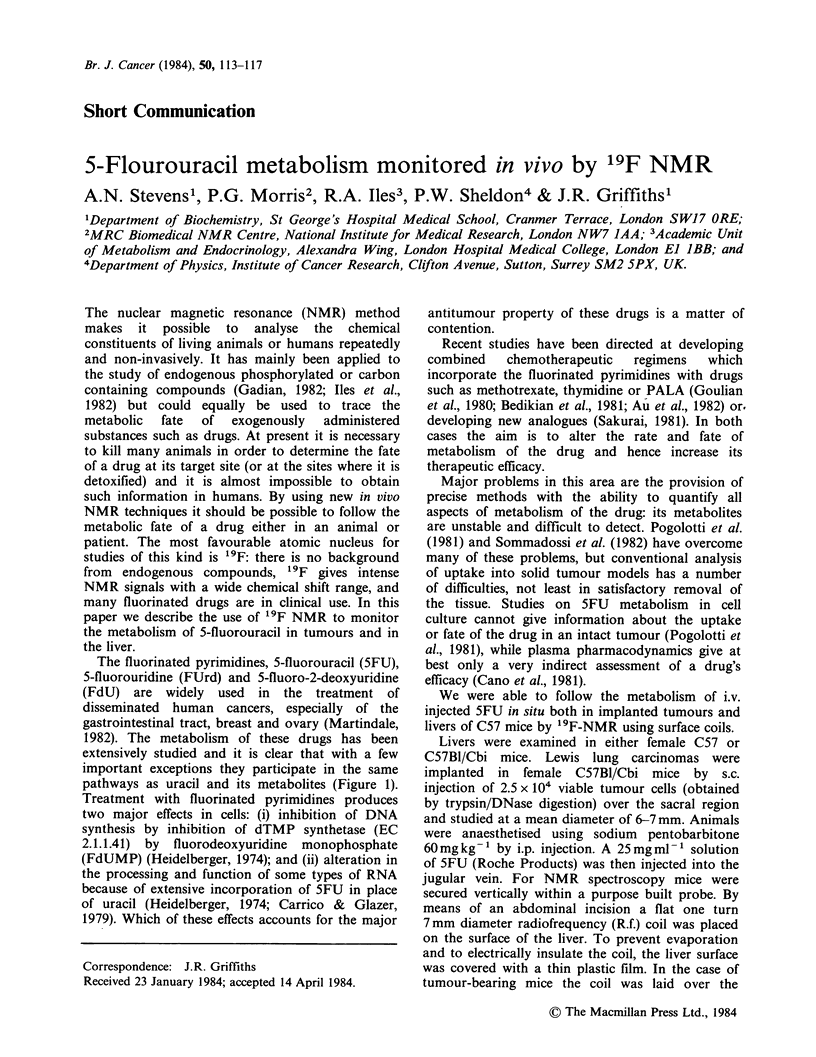

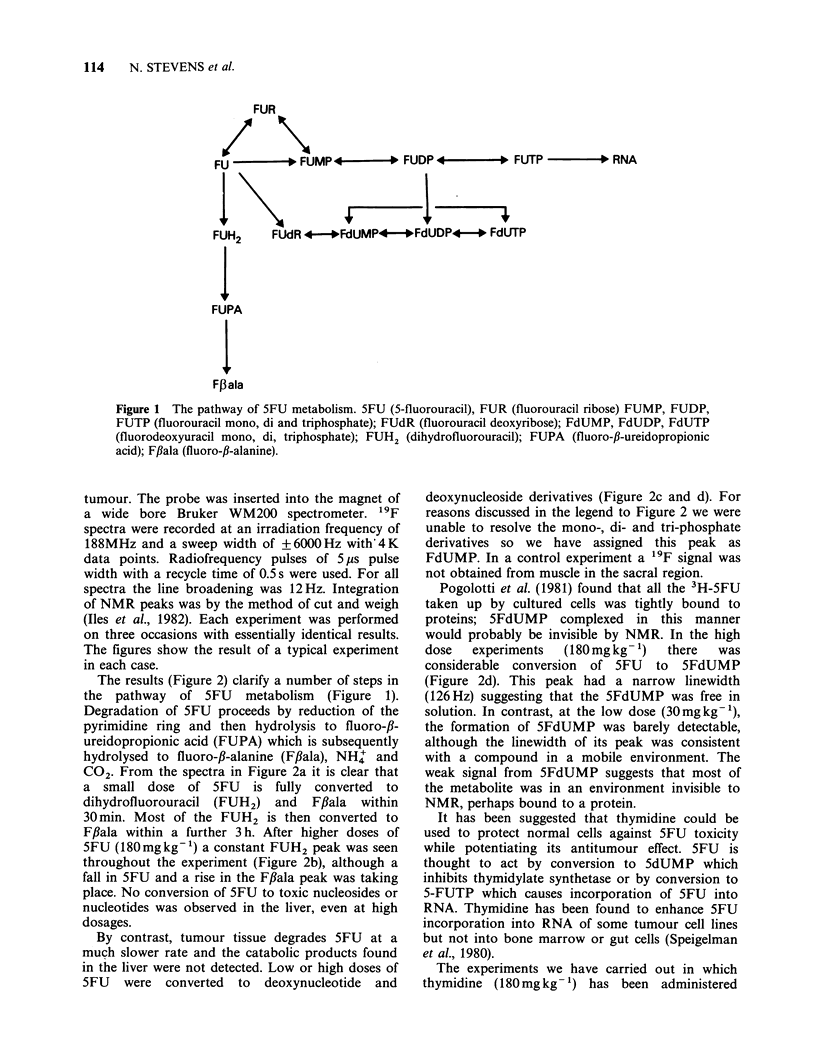

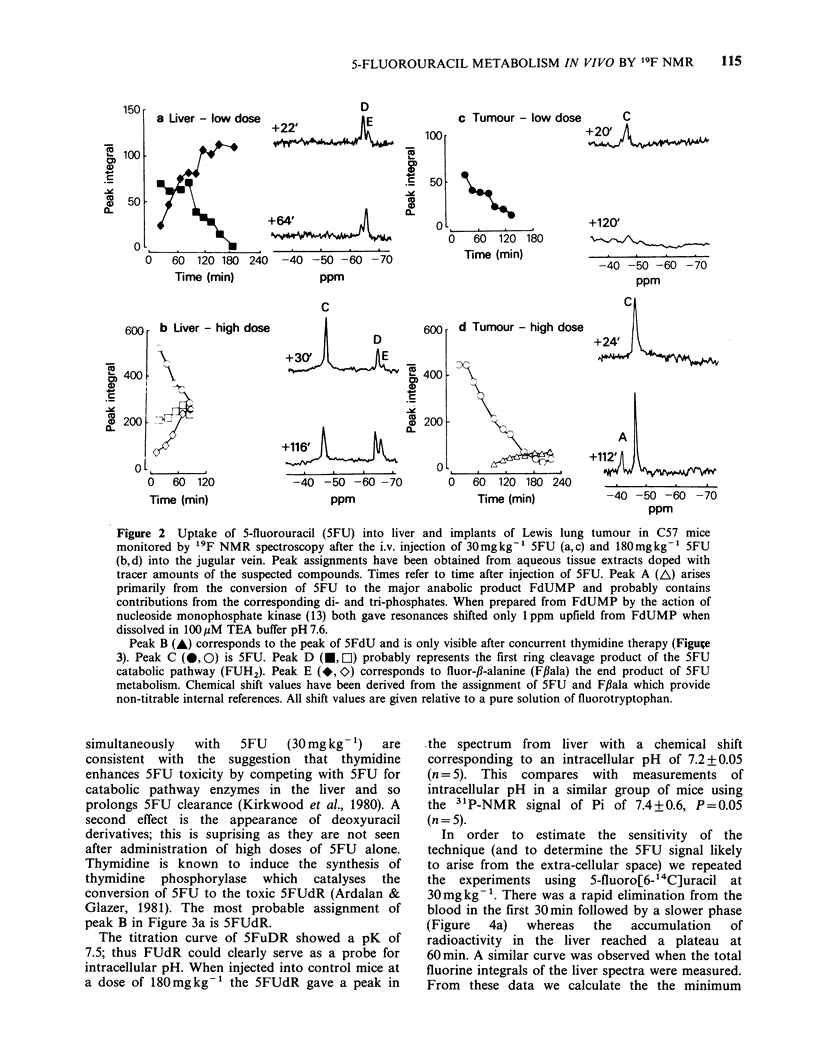

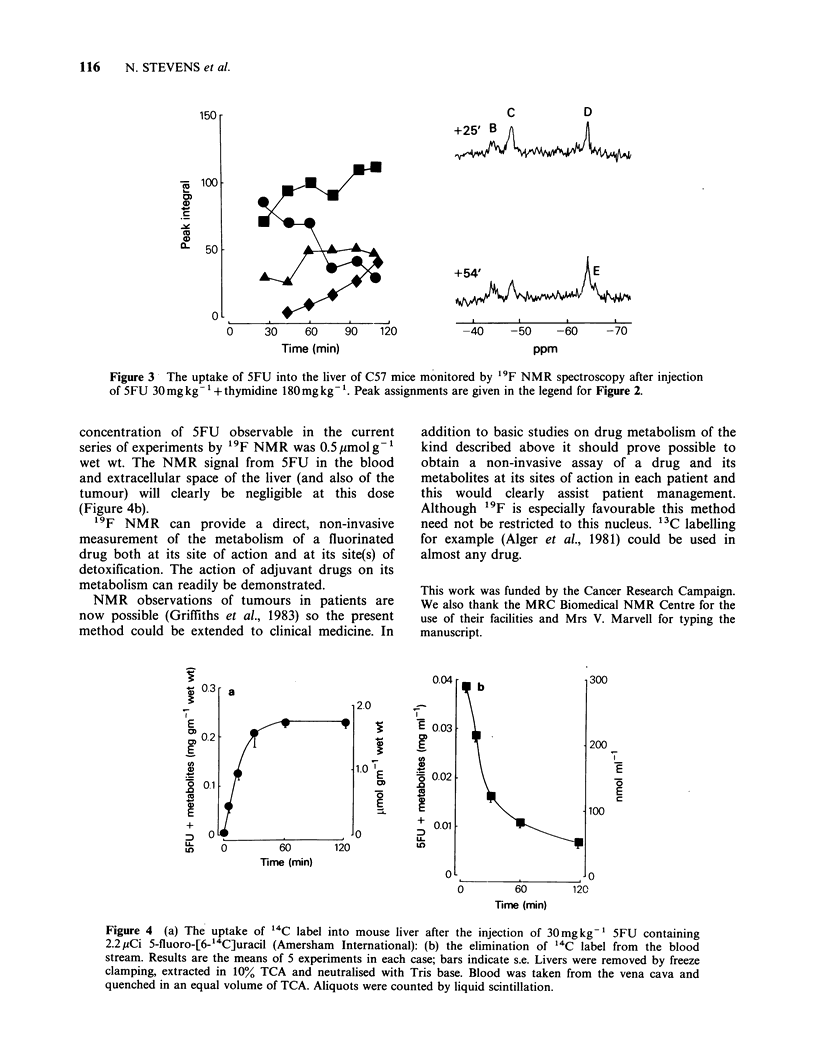

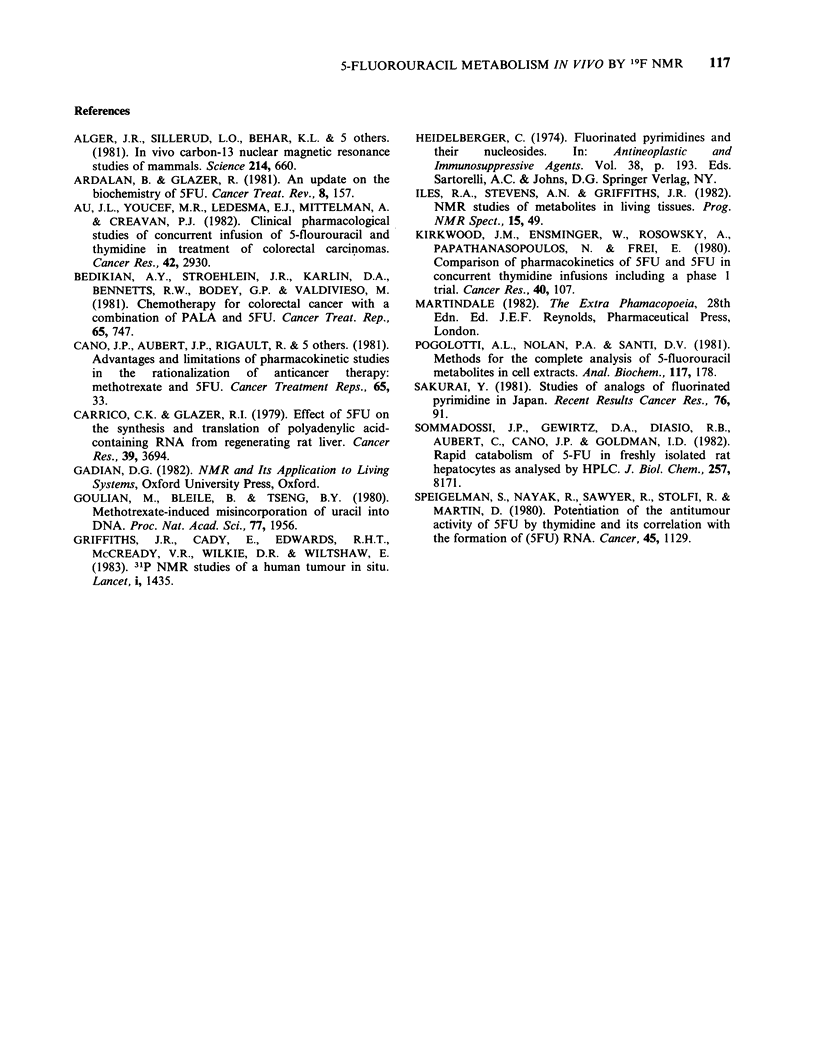


## References

[OCR_00469] Alger J. R., Sillerud L. O., Behar K. L., Gillies R. J., Shulman R. G., Gordon R. E., Shae D., Hanley P. E. (1981). In vivo carbon-13 nuclear magnetic resonance studies of mammals.. Science.

[OCR_00474] Ardalan B., Glazer R. (1981). An update on the biochemistry of 5-fluorouracil.. Cancer Treat Rev.

[OCR_00478] Au J. L., Rustum Y. M., Ledesma E. J., Mittelman A., Creaven P. J. (1982). Clinical pharmacological studies of concurrent infusion of 5-fluorouracil and thymidine in treatment of colorectal carcinomas.. Cancer Res.

[OCR_00485] Bedikian A. Y., Stroehlein J. R., Karlin D. A., Bennetts R. W., Bodey G. P., Valdivieso M. (1981). Chemotherapy for colorectal cancer with a combination of PALA and 5-FU.. Cancer Treat Rep.

[OCR_00499] Carrico C. K., Glazer R. I. (1979). Effect of 5-fluorouracil on the synthesis and translation of polyadenylic acid-containing RNA from regenerating rat liver.. Cancer Res.

[OCR_00509] Goulian M., Bleile B., Tseng B. Y. (1980). Methotrexate-induced misincorporation of uracil into DNA.. Proc Natl Acad Sci U S A.

[OCR_00514] Griffiths J. R., Cady E., Edwards R. H., McCready V. R., Wilkie D. R., Wiltshaw E. (1983). 31P-NMR studies of a human tumour in situ.. Lancet.

[OCR_00531] Kirkwood J. M., Ensminger W., Rosowsky A., Papathanasopoulos N., Frei E. (1980). Comparison of pharmacokinetics of 5-fluorouracil and 5-fluorouracil with concurrent thymidine infusions in a Phase I trial.. Cancer Res.

[OCR_00543] Pogolotti A. L., Nolan P. A., Santi D. V. (1981). Methods for the complete analysis of 5-fluorouracil metabolites in cell extracts.. Anal Biochem.

[OCR_00548] Sakurai Y. (1981). Studies of analogs of fluorinated pyrimidine in Japan.. Recent Results Cancer Res.

[OCR_00553] Sommadossi J. P., Gewirtz D. A., Diasio R. B., Aubert C., Cano J. P., Goldman I. D. (1982). Rapid catabolism of 5-fluorouracil in freshly isolated rat hepatocytes as analyzed by high performance liquid chromatography.. J Biol Chem.

[OCR_00560] Spiegelman S., Nayak R., Sawyer R., Stolfi R., Martin D. (1980). Potentiation of the anti-tumor activity of 5FU by thymidine and its correlation with the formation of (5FU)RNA.. Cancer.

